# A case series of a pav+ weaning protocol in an acute care environment demonstrating an absence of weaning failure

**DOI:** 10.1186/2197-425X-3-S1-A1008

**Published:** 2015-10-01

**Authors:** E Rohrs, S Reynolds, J Zurba

**Affiliations:** Fraser Health Authority, New Westminster, Canada; University of British Columbia, Vancouver, Canada; Department of Critical Care Medicine, Royal Columbian Hospital, New Westminster, Canada

## Introduction

Most patients undergoing mechanical ventilation (MV) in critical care units are liberated from the ventilator within a median of 7 days. Unfortunately, some patients experience difficulty in liberation from MV during their ICU stays and require extended time on MV. It has been estimated that 40% of the patient's duration on the ventilator is spent weaning.[1] Moreover, although patients requiring prolonged mechanical ventilation represent approximately 6% of all ventilated patients, they consume 37% of intensive care unit resources.[2] Therefore, accelerating the liberation of prolonged weaning patients from MV improves patient care and could yield significant economic benefits.

## Rationale

Which weaning strategy is “best” is controversial. Proportional Assist Ventilation (PAV+) has many aspects that are theoretically beneficial as part of a difficult weaning protocol.

## Methods

Single center case series of patients undergoing a PAV+ protocolized wean from an 18 bed mixed medical/surgical/ trauma Canadian tertiary care ICU.

## Results

Over the 39 month (3.25 year) period 46 patients underwent a slow wean, 40 of whom spent greater than 70% of their time weaning in a PAV+ mode. During weaning from MV there were 4/40 (10%) deaths in the PAV+ group, despite a high admitting APACHE II score. All patients in the PAV+ group who survived were successfully weaned from MV over a median of 13.3 (9.2-20) days.

## Conclusions

Although this study was conducted in an acute hospital context that is significantly different from Long Term Acute Care Hospitals, it raises the possibility that a PAV+ weaning protocol could be superior to PSV or t piece weaning strategies. Further studies are needed.

## Grant Acknowledgment

Study funded by grant from Covidien.

**Table 1:** Clinical Parameters of Wean Group vs. Literature.

	PAV + Cohort (n = 40)	Rose 2012(5) (n = 115)	Schönhofer 2002(6) (n = 403)	Jubran 2013a(7) (n = 152)	Jubran 2013b(7) (n = 160)	Pilcher 2005(8) (n = 153)	Scheinhorn 2002a(9) (n = 252)	Scheinhorn 2002b(9) (n = 238)
Age (years) median (IQR)*	69 (60.5-74.3)	70 (59-77)	66.0 (58.2-71.6)	70 (62-79)	70 (63-77)	70 (63-77)	73	71
Female Gender	48.00%	49.60%	35.50%	42%	50%	52%		
BMI	25.55 (20.3-31)		23.1 (19.6-27.6)					
APACHE II at admission	24 (17.8-28)	11.9 (+/- 4.1)*		15 (13-19)*	15 (14-15)*	15 (10-20)		
SOFA at ICU Admission	9 (8-12)			41 (30-62)				
Length of Hospital Stay (days) median (IQR)	68 (44.3-105)			39/152 (26%)	42 (28-63)		42.5	49
In hospital Mortality	15/40 (38%)		98/403 ( 24.3%)	14.50%	41/160 (26%)	42/153 (27%)		
Death during Weaning	4/40 (10%)	13%	24.30%	14.50%	10%	26.10%	30.70%	
Weaning Failure-Ventilatory Dependance	0	33.90%	19.00%	40.80%	36.90%	34.60%	17.90%	10.90%
